# Evolution and Adaptation of *Legionella pneumophila* to Manipulate the Ubiquitination Machinery of Its Amoebae and Mammalian Hosts

**DOI:** 10.3390/biom11010112

**Published:** 2021-01-15

**Authors:** Christopher T.D. Price, Yousef Abu Kwaik

**Affiliations:** 1 Department of Microbiology and Immunology, University of Louisville, Louisville, KY 40202, USA; christopher.price@louisville.edu; 2 Center for Predictive Medicine, College of Medicine, University of Louisville, Louisville, KY 40202, USA

**Keywords:** legionella pneumophila, ubiquitin, amoebae, F-box, U-box, E3-ubiquitin ligase, SNL E3-ligase, E1/2-independent ubiquitin ligase, deubiquitinase, Dot/Icm, effectors, evolution

## Abstract

The ubiquitin pathway is highly conserved across the eukaryotic domain of life and plays an essential role in a plethora of cellular processes. It is not surprising that many intracellular bacterial pathogens often target the essential host ubiquitin pathway. The intracellular bacterial pathogen *Legionella pneumophila* injects into the host cell cytosol multiple classes of classical and novel ubiquitin-modifying enzymes that modulate diverse ubiquitin-related processes in the host cell. Most of these pathogen-injected proteins, designated as effectors, mimic known E3-ubiquitin ligases through harboring F-box or U-box domains. The classical F-box effector, AnkB targets host proteins for K^48^-linked polyubiquitination, which leads to excessive proteasomal degradation that is required to generate adequate supplies of amino acids for metabolism of the pathogen. In contrast, the SidC and SdcA effectors share no structural similarity to known eukaryotic ligases despite having E3-ubiquitin ligase activity, suggesting that the number of E3-ligases in eukaryotes is under-represented. *L. pneumophila* also injects into the host many novel ubiquitin-modifying enzymes, which are the SidE family of effectors that catalyze phosphoribosyl-ubiquitination of serine residue of target proteins, independently of the canonical E1-2-3 enzymatic cascade. Interestingly, the environmental bacterium, *L. pneumophila,* has evolved within a diverse range of amoebal species, which serve as the natural hosts, while accidental transmission through contaminated aerosols can cause pneumonia in humans. Therefore, it is likely that the novel ubiquitin-modifying enzymes of *L. pneumophila* were acquired by the pathogen through interkingdom gene transfer from the diverse natural amoebal hosts. Furthermore, conservation of the ubiquitin pathway across eukaryotes has enabled these novel ubiquitin-modifying enzymes to function similarly in mammalian cells. Studies on the biological functions of these effectors are likely to reveal further novel ubiquitin biology and shed further lights on the evolution of ubiquitin.

## 1. Introduction

Eukaryotic cells utilize diverse post-translational modifications to exert various biological functions in the cell. These modifications include phosphorylation, hydroxylation, lipidation and the covalent linkage of small modifying proteins. Chief among these small modifying proteins is the 76-amino acid protein termed ubiquitin. The broad importance of ubiquitin in eukaryotic biology was highlighted in 2004, with the discovery being awarded the Nobel Prize for chemistry. Ubiquitin can be linked to proteins as a single monomer, termed monoubiquitination, or as a polymeric chain of ubiquitin moieties termed polyubiquitination [1,2,3]. Canonical ubiquitination involves three major steps, activation, conjugation and ligation, each of which are catalyzed by different groups of enzymes. These are E1 ubiquitin-activating enzymes, E2 ubiquitin-conjugating enzymes and E3 ubiquitin ligase enzymes, respectively [1,2,3]. Ubiquitin is initially covalently linked to lysine residues within the target proteins or alternatively cysteine, serine or threonine residues, or added to the amino group of the N-terminal amino acid [1]. For polyubiquitination, successive ubiquitin moieties are covalently linked to one of seven different lysine residues within the ubiquitin protein or the N-terminal methionine [1,2,3]. The most widely investigated form of polyubiquitination are K^48^-linkages [4]. The K^48^-linked polyubiquitination chain earmarks a target protein for degradation by the proteasome machinery, which cleaves the protein into small oligopeptides that are subsequently cleaved into individual amino acids by oligo- and amino-peptidases [1,4,5]. Recycling of proteins in eukaryotic cells is extremely important for normal cellular biology. Monoubiquitination and other forms of polyubiquitination regulate a plethora of functions in eukaryotic cells including vesicular traffic, DNA repair, apoptosis, immunity, autophagy, carcinogenesis, translation and inflammation [6,7,8].

A key to the success of intracellular bacterial pathogens is their evolution to evade the innate and adaptive immune responses. Just as important is that bacterial pathogens have evolved to modulate various biochemical and cellular functions in host cells to render them permissive for bacterial proliferation [9]. Intracellular pathogens have evolved to adapt to the intracellular environment within vacuoles that are not trafficked properly within the endosomal lysosomal degradation pathway and evade various aspects of the innate immune response [10,11,12]. The cellular pathways manipulated by pathogen are involved in numerous aspects of the host cell biology, such as signaling, autophagy, apoptosis and inflammasomes [13,14,15,16,17,18,19,20,21]. In addition, many intracellular bacterial pathogens have evolved to modulate and reprogram host metabolism to generate the nutrients needed to support pathogen proliferation [22,23,24,25]. A major theme among pathogens that have evolved to modulate cellular biology is the injection of bacterial proteins directly into the host cell through specialized nanosyringes that penetrate eukaryotic membranes and inject pathogenic effector proteins, and these nanosyringes are classified as type III-IX secretions systems [26,27,28,29,30,31]. Many host post-translational modifications machineries are targeted by various injected bacterial effectors [32]. Since a plethora of eukaryotic cellular processes are regulated by ubiquitination, it is not surprising that intracellular pathogens have evolved mechanisms to hijack host ubiquitination pathways to rewire host cell processes. Multiple intracellular pathogens including *Salmonella*, *Mycobacterium*, *Shigella*, *Coxiella*, *Anaplasma*, *Chlamydia* and *Legionella* manipulate the host ubiquitin pathway to evade host restriction and generate a replicative niche in eukaryotic cells [33,34,35,36,37,38,39,40,41]. However, amongst these intracellular pathogens, *L. pneumophila* possesses the most diverse and novel array of mechanisms to manipulate the eukaryotic ubiquitination machinery [42]. *L. pneumophila* resides in a diverse array of amoebal hosts, and as such, has evolved with a complex toolbox of effector proteins to adapt and survive in these environmental unicellular hosts [43,44,45].

*L. pneumophila* is primarily found in aquatic environments and has evolved to invade and proliferate within diverse amoebae species [46], but upon transmission to humans, causes pneumonia [43,47,48,49]. This bacterium has evolved to bypass the default endosomal–lysosomal pathway within eukaryotic cells and generates a replicative vacuole derived from the endoplasmic reticulum, termed the *Legionella*-containing vacuole, (LCV) [50,51,52,53]. Upon uptake by coiling phagocytosis by host cells [54], this bacterium immediately begins to modulate a plethora of host cell processes through injection of a complex tool box of over 320 different effector proteins through the Dot/Icm type IVB secretion system, which functions as a nanosyringe [55,56]. Most effectors are injected following uptake into the host cells, but some such as AnkB have been shown to be injected directly upon cell contact prior to phagocytosis [57]. This large cache of effectors enables *L. pneumophila* to be a generalist pathogen in terms of host range and survive within a wide range of hosts in the dynamic natural environment, unlike other intracellular pathogens, which have much smaller repertoires of effectors, reflecting their limited host range [43]. The adaptation of *L. pneumophila* to diverse amoebal species in aquatic environments and the widespread development of man-made water systems that increase aerosol delivery of contaminated water droplets, has led to this organism becoming an accidental pathogen in humans, causing an atypical pneumonia called Legionnaires’ disease [58,59,60]. When *L. pneumophila* reaches alveolar macrophages in the lungs, this organism invades the alveolar macrophages and targets cellular pathways similar to that during infection of its natural amoebae hosts [43,51]. Interestingly, humans are a dead-end host for *L. pneumophila*, since there is no person-to-person transmission of the bacteria. Therefore, the *L. pneumophila* effectors have been subjected to natural selection pressure to be amoebae-adapted only, during coevolution [43]. However, due to strong conservation of numerous eukaryotic pathways such as endocytic trafficking and ubiquitination, many of these effectors often function similarly in higher eukaryotes including humans, though the outcomes may diverge. It is not surprising that numerous effectors of *L. pneumophila* harbor eukaryotic-like domains that enable them to mimic eukaryotic functions, and these likely have been acquired by *L. pneumophila* from the amoebal host via interkingdom horizontal gene transfer [43,44,45].

In this review, diverse manipulations of the eukaryotic ubiquitination pathways by *L. pneumophila* effectors will be explored. This organism possesses several effectors that harbor eukaryotic domains that target different aspects of the ubiquitination pathway, and additionally some effectors have shed light on novel ubiquitination chemistry that was previously unknown, and these novel ubiquitinations are likely present in unicellular eukaryotes. Furthermore, since *L. pneumophila* effectors are likely amoebae-adapted, possible functions of these effectors in the natural amoebae-host will be discussed.

## 2. F-Box Effectors

*L. pneumophila* harbors at least five effectors that harbor the eukaryotic F-box domain [57,61,62]. In humans there are at least 70 F-box proteins [63], indicating that regulation of protein ubiquitination by the SCF ligase machinery is a key mechanism controlling protein fate in eukaryotic cells. In eukaryotes, F-box proteins form part of a multi-protein complex designated the E3 SCF-ubiquitin ligase [63]. The SCF-ligase complex is composed of a RING-domain protein RING-box 1 (RBX1), cullin 1 (CUL1), S-phase-kinase associated protein 1 (SKP1) and a protein harboring an F-box domain, that directly binds SKP1 [63]. The associated F-box protein determines the specificity of the ubiquitination event by binding to target proteins through protein–protein interaction domains. [63]. In higher eukaryotes, F-box proteins are classified into three subfamilies on the basis of the protein–protein interaction domains they possess and include; those with leucine-rich repeats (FBXL), WD40 repeats (FBXW) or uncharacterized domains (FBXO) [64]. In contrast, F-box proteins in lower eukaryotes such as *Acanthamoebae*, can possess other protein–protein binding domains such as the ankyrin repeat [65].

**Figure 1:**
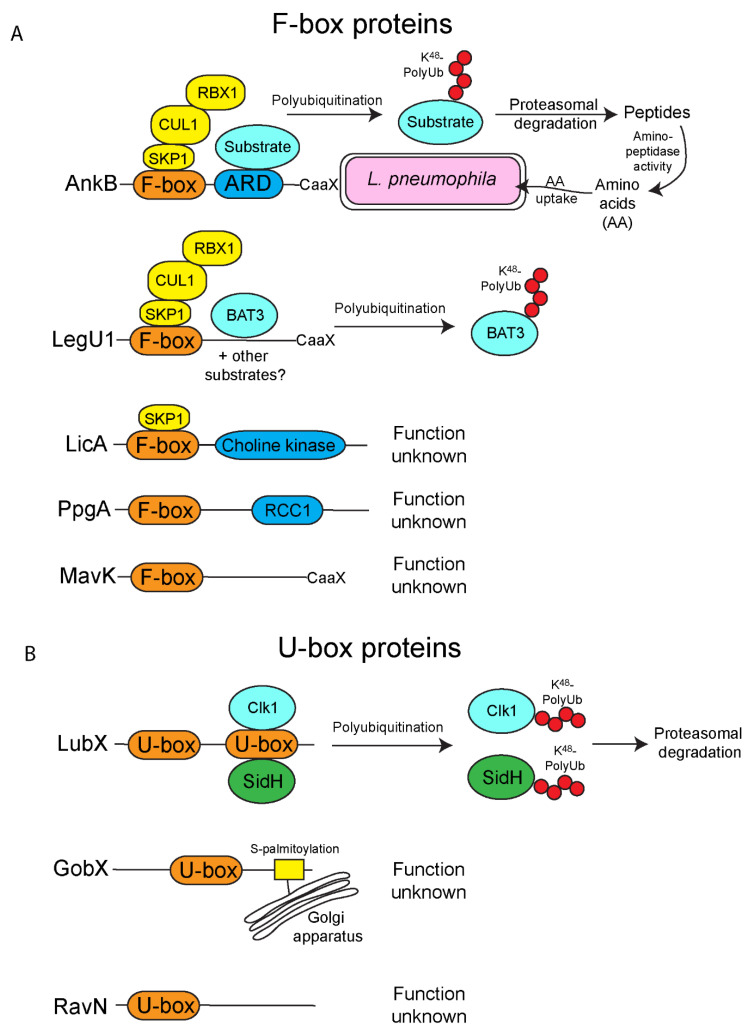
The “classical” E3-ubiquitin ligases of *L. pneumophila*. *L. pneumophila* has multiple effectors that show homology to the classical F-box and U-box families that are widespread in eukaryotes. (**A**) F-box effectors of *L. pneumophila*. There are five known *L. pneumophila* F-box effectors. Firstly, AnkB, which harbors an F-box, an ankyrin repeat domain (ARD) and a C-terminal CaaX motif that is targeted for farnesylation, enabling localization to the *Legionella*-containing vacuole, (LCV) membrane. The F-box domain of AnkB interacts with the host Skp1-Cul1 (SCF) complex to promote the decoration of the LCV with K^48^-linked polyubiquitinated proteins. These proteins are ultimately degraded by the host proteasomes to generate copious quantities of amino acids required for metabolism of *L. pneumophila*. LegU1 also interacts with the SCF complex and directs the polyubiquitination of the host protein, BAT3. It is possible LegU1 also targets other as yet unidentified host proteins. Similar to AnkB, LegU1 also has a C-terminal CaaX motif, targeting this effector to host membranes. LicA interacts with the Skp1 component of the SCF complex but not Cul1, suggesting a LicA forms a non-canonical complex, and the functional role of this effector is unknown. The functional roles of MavK, which also has a C-terminal CaaX motif and PpgA are unknown. (**B**) U-box effectors of *L. pneumophila*. *L. pneumophila* injects three effectors with the U-box domain. LubX ubiquitinates the host factor Clk1. Additionally, LubX functions as a metaeffector and directs ubiquitination of the injected effector, SidH, which is then targeted for proteasomal-mediation degradation. GobX is targeted to the Golgi apparatus where it is embedded into the membrane through S-palmitoylation modification, however the functional role of GobX is unknown. RavN also harbors the U-box domain, but the role of this effector is unknown.

The ankyrin B (AnkB/LegAU13/Lpg2144) effector of *L. pneumophila* plays a fundamental role in the biology of this organism during infection of both its natural amoebae host and the accidental human host. Unlike most effectors in *L. pneumophila*, AnkB is essential for efficient replication of this organism in both amoebae, human cells and a mouse model of pulmonary disease [57,61,66,67]. AnkB possesses an N-terminal F-box domain and an ankyrin repeat domain (ARD) that is comprised of three ankyrin repeats (Figure 1A) [57,61,68]. The AnkB effector functions as a canonical eukaryotic F-box protein. Both X-ray crystallography and biochemical assays show this protein interacts with the host SKP1 protein, similar to other F-box proteins in eukaryotes (Figure 1A) [23,57,68]. *L. pneumophila* exclusively uses amino acids as its main source of carbon and energy. However, *L. pneumophila* is auxotrophic for several amino acids and therefore, this organism must acquire amino acids directly from the host cell [69]. AnkB is responsible for dense accumulation of K^48^-linked polyubiquitinated proteins around the LCV [23,57,66] that are destined for degradation by the proteasome in both amoebae and human macrophages [23]. AnkB-mediated protein turnover, through hijacking the E3-SCF complex, ultimately provides an ample supply of free amino acids for *L. pneumophila* metabolism (Figure 1A). Despite AnkB having a clear role during infection of host cells, specific proteins that are targeted by this effector for K^48^-linked polyubiquitination have yet to be determined.

Besides mimicking eukaryotic F-box proteins to specifically polyubiquitinate target host proteins, AnkB also undergoes eukaryotic post-translational modifications. Firstly, injection of AnkB into the host cell by the Dot/Icm type IV secretion system results in exclusive targeting of the effector into the LCV membrane [67] and this is mediated by the host farnesylation machinery, that modifies the CaaX motif located at the C-terminus of the protein (Figure 1A) [67]. Farnesylation is a highly conserved post-translation lipid modification of eukaryotic proteins that confers hydrophobicity on the modified protein, and enables its targeting to membranes by embedding into the lipid bilayer [70]. Localized accumulation of AnkB at the LCV is required for the function of this effector (Figure 1A) [67]. Interestingly, in some strains of *L. pneumophila*, there is a frame shift mutation at the 3′ end of the gene, which generates a premature stop codon, abolishing the sequence of the C-terminal CaaX motif [66,71]. However, the frameshift mutation generates an ER retention motif that still enables AnkB to embed into the ER-derived LCV membrane, illustrating the strong selective pressure to maintain AnkB at the LCV outer leaflet and the localized K^48^-linked polyubiquitination and protein turnover required for bacterial metabolism [71]. Secondly, the first ankyrin repeat in the ARD of AnkB undergoes asparaginyl hydroxylation via the activity of the FIH1 enzyme, and this modification is required for the optimal activity of AnkB during infection [72]. Asparaginyl hydroxylation can act as an on/off switch for protein function and may also enhance protein/protein interactions [73]. Additionally, AnkB is modified by K^11^-linked polyubiquitination independent of its F-box domain during infection of host cells, though the functional consequence of this is yet to be determined [74].

In higher eukaryotes, such as humans, F-box proteins do not contain ARD regions, but utilize other protein/protein interaction motifs such as the leucine rich repeat and WD40 domains [64]. Bioinformatic analyses show however, that in amoebal species, F-box proteins can contain ARD regions [65]. Since *L. pneumophila* has evolved within multiple species of amoebae in the natural environment, it is more likely that AnkB has been acquired through interkingdom gene transfer from the amoebal hosts.

While AnkB is the best characterized F-box protein found of *L. pneumophila*, this organism has at least four other F-box proteins that are injected into host cells by the Dot/Icm system [62] (Figure 1). Unlike AnkB, which is found in all sequenced *L. pneumophila* strains, the other F-box proteins are found only in some strains of *L. pneumophila*. Of these four, PpgA (Lpg2224) and MavK (Lpg2525), which are only found in some strains, do not associate with components of the host ubiquitination machinery, and may therefore play a different role during infection, and target proteins found in diverse unicellular amoebal hosts of *L. pneumophila* (Figure 1A) [62]. Interestingly, PpgA shares 25.4% amino acid similarity to another effector, LegG1/Lpg1976, which functions as a bacterial Ran activator to promote microtubule stabilization [75]. Therefore, it is possible PpgA plays an alternative role similar to LegG1, which is distinct from classical F-box proteins. Since these studies were only performed in mammalian cells, it is possible that PpgA and MavK interact with amoebae-specific ubiquitination steps, the host cells with which these effectors have been adapted too. LicA (Lpg1408), which is found in all the sequenced *L. pneumophila* strains, interacts with SKP1, but not CUL1 (Figure 1A) [62]. This suggests that this protein may form a non-canonical SCF complex containing a different cullin [62]. Again, since these studies utilize mammalian cells, and not the natural amoebal host, further work is needed to confirm that LicA does indeed interact with SKP1 to form a canonical or non-canonical SCF complex. LegU1 (Lpg0171) interacts with both SKP1 and CUL1, forming an active E3 ubiquitin ligase complex, and is found in most sequenced *L. pneumophila* genomes [62]. The LegU1 effector directs the ubiquitination of the human protein BAT3 [62], a chaperone protein that plays key roles in modulating apoptosis, ER stress, p53-regulated expression and Hsp70 stability (Figure 1A) [76]. It is not currently clear what the exact role of LegU1-mediated BAT3 ubiquitination is during the course of infection by *L. pneumophila*. It is important to note that the *Amoebozoa* genera, the primary hosts of *L. pneumophila* do not have a close homologue of the mammalian BAT3 protein, suggesting that the real target of LegU1 has yet to be determined and that maybe BAT3 is an accidental mammalian interaction partner of this effector. Interestingly, both MavK and LegU1 have putative CaaX farnesylation motifs, similar to AnkB (Figure 1A) [67,77], and it will be interesting to determine if farnesylation is crucial for their membrane anchoring and biological activity following their injection into the host cell.

To date, the exact host proteins targeted by any of the *L. pneumophila* F-box proteins in the natural amoebal hosts remain elusive. For AnkB, no specific targets have been identified despite multiple efforts to do so in both mammalian and amoebal cells, and this may reflect a broad or temporal interaction range for this effector, which reflects its function to generate copious amino acids for the bacteria regardless of which host the bacterium has invaded. For the other four F-box proteins, studies have been limited to the role of these proteins in mammalian cells, not the amoebal host that these proteins have been evolutionarily adapted to. Future studies will need to focus on the role of these effectors, and the proteins they interact with during infection of the natural amoebae host. This will provide valuable information on how *L. pneumophila* survives within predatory cells such as amoebae and macrophages, and may undercover novel functions of F-box proteins in general.

## 3. U-Box Effectors

Apart from harboring effectors with the F-box domain, *L. pneumophila* also harbors three effectors that contain the U-box E3-ubiquitin ligase domain (Figure 1B) [78,79,80,81]. Unlike the multisubunit SCF E3-ubiquitin ligase, proteins with the U-box E3-ubiquitin ligase domain interact alone with E2-conjugating enzymes to ubiquitinate target proteins [82]. LubX, (Lpg2830), was the first bacterial effector identified harboring the eukaryotic U-box domain [78,79]. Curiously, LubX harbors two U-box domains and was shown to interact with a diverse group of mammalian E2-conjugating enzymes including UBE2W, UBEL6, and members of the UBE2D and UBE2E families to direct ubiquitination of mammalian Cdc2-like kinase 1 (Clk1) [78,83]. LubX-mediated ubiquitination of Clk1 results in degradation of this protein by the proteasome, however, the outcome of Clk1 degradation during *L. pneumophila* is still unclear (Figure 1B) [78]. Even though LubX has two U-box domains, X-ray crystallography of the protein structure revealed that only the N-terminal U-box retains features that recognize E2-conjugating enzymes while the C-terminal U-box has diverged and does not appear to interact with E2-conjugating enzymes of mammals [83]. It is possible this domain is relevant in the unicellular amoebal hosts of *L. pneumophila*. Unlike LegU1, where its interacting partner in mammalian cells, BAT3, is absent in amoebae, homologs of Clk1 are widespread in the natural amoebal hosts of *L. pneumophila*, suggesting ubiquitination of Clk1 by LubX is likely a specific conserved function of this effector. It will be important to determine if amoebal homologues of the mammalian Clk1 are ubiquitinated by LubX, similar to what has been shown in mammalian cells. Recently, it has come to light that some *L. pneumophila* effectors do not target host cell proteins, but can modulate other effectors injected into the host cell by the bacterium, and are termed metaeffectors Distinct from LubX directing the ubiquitination of mammalian Clk1, LubX also functions as a metaeffector of the *L. pneumophila* effector, SidH (Lpg2829), and this may represent the true evolutionary and biological role of LubX in the amoebal host (Figure 1B) [79,84]. SidH is translocated early during infection by *L. pneumophila*, and when expressed in yeast, is toxic, but the role of this effector is unknown [79]. LubX, which accumulates throughout *L. pneumophila* infection, acts as a negative temporal regulator of SidH by ubiquitinating this protein, which is subsequently targeted for proteasomal degradation [79]. This demonstrates an exquisite manipulation of eukaryotic cell function of LubX by mimicking an E3-ligase to fine tune the turnover of another injected effector protein. To date, no other eukaryotic or *L. pneumophila* effectors are known to be targeted by LubX during infection.

The Golgi-localized effector, GobX (Lpg2455), also harbors a U-box domain and has been shown to have E3-ligase activity (Figure 1B) [81]. To date, specific host targets of GobX have not been identified and deletion of this effector from the *L. pneumophila* genome results in no discernable phenotype in mammalian cells [81]. Interestingly, GobX is specifically targeted to the host Golgi apparatus through exploitation of host S-palmitoylation lipidation (Figure 1B) [81]. Since there is no discernable role of GobX in mammalian cells, it is possible this effector targets a function in amoebal cells that either is divergent from or not present in higher eukaryotes. It will be important to examine the role of GobX in the context of infection in the natural amoebal host, and this may uncover novel manipulations of the eukaryotic ubiquitination machinery.

RavN (Lpg1111) was recently identified as a bona fide E3 ubiquitin ligase of *L. pneumophila* [80]. However, unlike the LubX and GobX effectors, BLAST analysis of the RavN primary amino acid did not reveal homology to known E3 ligases. Examination of the protein structure by X-ray crystallography revealed that the N-terminal region of the protein, which confers E3 ligase activity, has a U-box-like motif that interacts with E2-conjugating enzymes (Figure 1B) [80]. Like GobX, the target proteins of RavN and the biological role of this protein are currently unknown (Figure 1) [80]. It is likely that concerted studies using the natural amoebal host to uncover the target and role of this host-adapted effector will provide novel insight on both *L. pneumophila* and ubiquitin biology and its evolution. Additionally, since the primary amino acid sequence of GobX does not match any known eukaryotic E3 ligases, but shows functional homology to U-box proteins, it suggests that there might be a larger pool of *L. pneumophila* effectors that function as ubiquitin ligases. Furthermore, many ubiquitin ligases in eukaryotes may yet to be discovered that have conserved E3-ligase folds despite not matching well at the primary amino acid sequence level, expanding the ubiquitination machinery toolkit. Alternatively, it is also possible that *L, pneumophila*-specific ubiquitin ligases have evolved independently from eukaryotic enzymes, to perform similar functions in host cells.

## 4. E3-Ubiquitin Ligase Activity of SidC/SdcA

**Figure 2:**
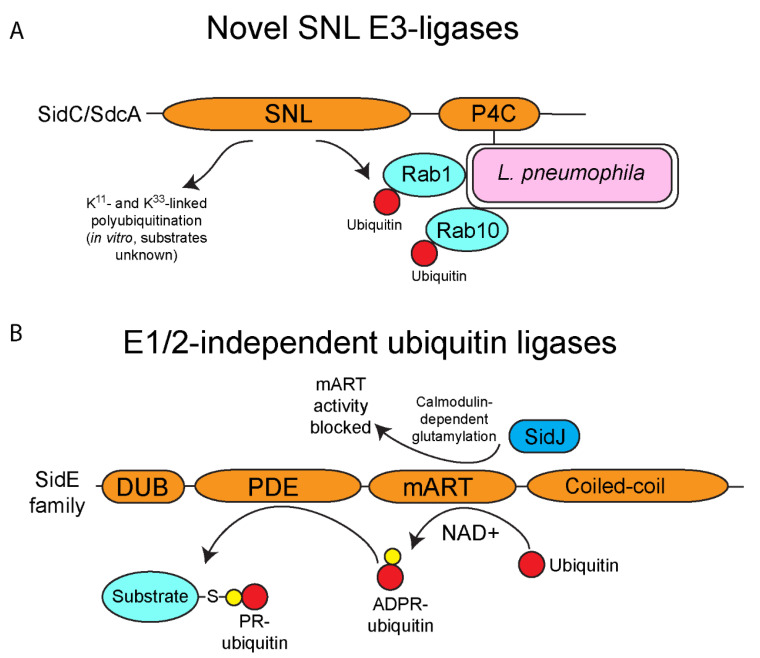
Novel ubiquitin ligases of *L. pneumophila*. *L. pneumophila* possesses multiple effectors that exhibit ubiquitin ligase activity but do not show any homology to any known eukaryotic ligases. (**A**) Novel SNL E3-ligases of *L. pneumophila*. *L. pneumophila* injects two paralogous effectors (SidC and SdcA) that possess the SidC N-terminal ubiquitin ligase (SNL) domain, which catalyzes K^11^- and K^33^-linked polyubiquitin linkages (in vitro), and direct monoubiquitination of host Rab1 and Rab10. Both effectors associate with the outer leaflet of the LCV membrane via their C-terminal PI4P binding domain. SidC/SdcA contribute to ER-derived vesicle recruitment to the LCV. The SNL domain has not been found in eukaryotic ubiquitin ligases. (**B**) E1/2-independent ubiquitin ligases of *L. pneumophila*. The E1/2-independent ubiquitin ligases of the SidE family are the first known ubiquitin ligases that function completely independently of the canonical activity of the E1 and E2 enzymes. The mART domain of the SidE family catalyzes the formation of ADP-ribosylated ubiquitin using NAD+ as a substrate. The PDE domain of the SidE family can then use ADP-ribosylated ubiquitin to add phosphoribosylated-ubiquitin to serine residues to substrate proteins, such as host Rab proteins and Rtn4. Activity of the SidE family is regulated by the metaeffector, SidJ, which catalyzes calmodulin-dependent glutamylation of the mART domain, and this blocks activity of this domain.

The effector SidC (Lpg2511) and its paralog, SdcA (Lpg2510) of *L. pneumophila*, anchor to the cytoplasmic leaflet of the LCV membrane through interaction of its C-terminal phosphatidylinositol-4-phosphate binding domain and mediates recruitment of ER-derived vesicles to the LCV (Figure 2A) [85,86,87,88,89]. Analyses of the SidC and SdcA structures by X-ray crystallography revealed an N-terminal fold that harbored a Cys-His-Asp catalytic triad typical of cysteine proteases and deubiquitinases, but this domain did not match any known protein structure [90,91]. A combination of ectopic expression in mammalian cells and in vitro ubiquitination assays confirmed that the N-terminal domain confers E3-ubiquitin ligase activity upon SidC [90]. This domain was termed the SidC N-terminal ubiquitin ligase (SNL) and preferentially catalyzes K^11^ and K^33^ polyubiquitin linkages in vitro (Figure 2A) [89,90]. Inactivation of the SNL domain of SidC impairs recruitment of ER-derived vesicles to the LCV membrane, indicating that the E3-ubiquitin ligase activity is important for this process, though the exact mechanism is unknown (Figure 2) [89,90]. To date, SidC and SdcA are known to target monoubiquitination of two small GTPases involved in vesicular fusion, Rab1 and Rab10 (Figure 2A) [87,92]. SidC and SdcA show E3-ubiquitin ligase activity despite having no structural homology to known eukaryotic ligases. Since new ubiquitin ligases have been identified with no match to “canonical” enzymes, it suggests that there are likely many undiscovered enzymes with novel E3-ubiquitin ligase folds in eukaryotes and potentially in *L. pneumophila* and other intracellular bacterial pathogens.

## 5. One Enzyme-Mediated Phosphoribosyl-Ubiquitination by the SidE Family Effectors

**Figure 3:**
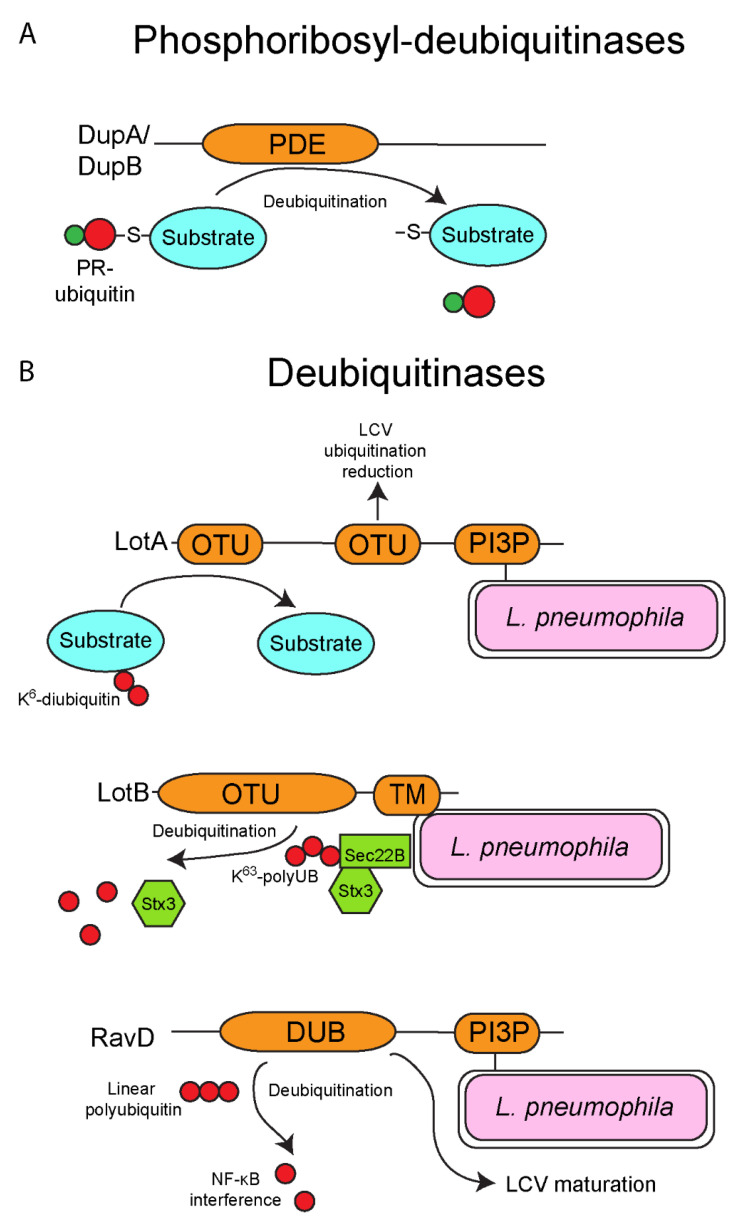
Deubiquitinases of *L. pneumophila*. Besides possessing several ubiquitin ligase, *L. pneumophila* also injects into the cytosol of host cells several deubiquitinases. (**A**) Phosphoribosyl-deubiquitinases of *L. pneumophila*. The SidE family-dependent phosphoribosyl-ubiquitination of proteins can be reversed by the deubiquitinase activity of the two injected effectors, DupA and DupB. Both DupA and DupB possess a PDE domain homologous to those found in the SidE family, but in contrast these domains catalyze the removal of phosphoribosyl-ubiquitin moieties from substrate proteins. (**B**) Deubiquitinases of *L. pneumophila*. *L. pneumophila* also possesses several deubiquitinases that specifically remove ubiquitin moieties from targeted proteins. LotA harbors two OTU domains and is localized to the LCV outer leaflet via its C-terminal PI3P-binding domain. LotA has been shown to reduce the overall ubiquitination of the LCV, and additionally specifically deubiquitinate proteins that are modified with a K^6^-diubiquitin moiety, though the functional importance of this effector is unknown. Similar to LotA, LotB also harbors an OTU domain and has a C-terminal transmembrane domain to allow localization to the LCV membrane. LotB specifically deubiquitinates K^63^-linked polyubiquitin chains from the host SNARE, Sec22B, resulting in the release of SNARE, Stx3. Finally, RavD harbors a deubiquitinase domain that specifically deubiquitinates linear polyubiquitin chains, and this may disrupt NF-κB signaling. Similar to LotA, RavD has the C-terminal PI3P-binding domain to allow localization of the effector of the LCV membrane.

The SidE family of *L. pneumophila* effectors is composed of four orthologous members, (SidE/Lpg0234, SdeA/Lpg2157, SdeB/Lpg2156 and SdeC/Lpg2153) and surprisingly, this family catalyzes ubiquitination of host proteins using a novel chemistry previously unknown in ubiquitin biology (Figure 2B). Canonically, ubiquitination in eukaryotes requires the sequential activity of the E1, E2 and E3 enzymatic cascade. However, the SidE family bypasses this requirement completely via the sequential activity of its mono-ADP-ribosyltransferase (mART) and phosphodiesterase domains (PDE). The mART domain catalyzes the transfer of ADP-ribose to the arginine 42 of ubiquitin using NAD+ as a substrate, generating ADPR-ubiquitin. ADPR-ubiquitin can then be used by the PDE domain to conjugate ubiquitin to serine residues on substrate proteins, generating a phosphoribosyl-ubiquitinated protein product (Figure 2B) [93,94,95,96,97,98,99,100]. The novel ubiquitination catalyzed by the SidE family appears to have multiple targets in the host cell including several Rab proteins and reticulon 4 to control tubular endoplasmic reticulum (ER) dynamics [93,100]. Interestingly, expression of the SidE family in yeast results in cellular toxicity, but this toxicity is reversed by the metaeffector, SidJ (Lpg2155) [101,102]. SidJ catalyzes calmodulin-dependent glutamylation of the mART domain of the SidE family, which blocks the ubiquitin ligase activity of these proteins (Figure 2B) [103,104,105,106,107]. Furthermore, recent studies identified two *L. pneumophila* effectors termed DupA (Lpg2154) and DupB (Lpg2509) that possess a phosphodiesterase (PDE) domain similar to the SidE family (Figure 3A) [108,109]. Unlike the SidE family, DupA and DupB specifically remove phosphoribosylated-ubiquitin conjugates from target proteins, acting as deubiquitinases (Figure 3A) [108,109]. Using a catalytically inactive DupA revealed that over 180 host proteins are phosphoribosyl-ubiquitinated by the SidE family, and these proteins are generally associated with ER fragmentation and recruitment of membrane to the LCV [109]. The combined activity of SidJ to block the SidE family catalytic activity and the presence of DupA and DupB to subsequently fine tune phosphoribosyl-ubiquitination suggests that during infection of host cells there is exquisite and complex control of the action of the SidE family. Furthermore, since *L. pneumophila* is adapted to its natural amoebal hosts, and that many effectors have been acquired through interkingdom gene transfer, it is likely that phosphoribosyl-ubiquitination is widespread in lower unicellular eukaryotes, further increasing the complexity of ubiquitin biology and its evolution in mammals.

## 6. Deubiquitinases

In addition to harboring multiple ubiquitin ligases and the two deubiquitinases DupA and DupB described above, *L. pneumophila* possesses a number of other effectors that act as deubiquitinases, which catalytically cleave ubiquitin moieties off target proteins, adding further complexity to the pathogen/host ubiquitination manipulation. LotA (Lpg2248/Lem21) harbors two eukaryotic like ovarian tumor (OTU) superfamily domains, a domain that exhibits cysteine protease activity and function as deubiquitinases (Figure 3B) [110]. LotA is localized to the outer leaflet of the LCV membrane through interaction of its phosphatidylinositol 3-phosphate binding region, and similar to AnkB albeit through a different mechanism [67], membrane localization is required for LotA function (Figure 3B) [110]. Interestingly, the two OTU domains of LotA appear to have different roles during infection of cells. The second OTU reduces overall ubiquitination of the LCV, while the first OTU specifically targets K^6^-linked diubiquitin moieties [110], though the functional consequences of LotA deubiquitination is unknown (Figure 3B) [110]. Deletion of *lotA* does not impair intracellular growth of *L. pneumophila*, at least in mouse bone marrow-derived macrophages (bMDMs), however deletion of *lotA* in combination with the SidE family of effectors further diminishes survival of *L. pneumophila* in mouse bMDMs [110]. The functional role and protein targets of LotA during infection of host cells is currently unknown, and like many of the *L. pneumophila* effectors that manipulate the eukaryotic ubiquitination system, analysis of the role of LotA during infection of the natural amoebae host will be crucial to unravel the exact function of this effector. Another effector, LotB (Lpg1621/Ceg23), also harbors an OTU domain and deubiquitinates K^63^-linked polyubiquitin chains at the LCV (Figure 3B) [111,112]. Unlike LotA, LotB has a clear host target resulting in modulation of the early secretory pathway [112]. Membrane fusion in eukaryotes requires the pairing of proteins called soluble N-ethylmaleimide-sensitive factor attachment protein receptors (SNAREs) [113]. The host vesicular SNARE, Sec22B is recruited to the LCV and binds to the t-SNARE, Stx3 to help establish the early LCV membrane [114,115,116]. At the LCV, Sec22b is ubiquitinated with K^63^-linked polyubiquitinated chains and this is dependent on effector translocation by *L. pneumophila* [112]. LotB specifically deubiquitinates the K^63^-linked polyubiquitinated chains from Sec22b at the LCV, resulting in disassociation of Stx3 from Sec22b (Figure 3B) [112]. Even though LotB has a clear host cell target, the overall functional consequence of its activity, at least in mammalian cells is unclear, since deletion of the *lotB* gene does not impair intracellular replication of *L. pneumophila* and formation of the LCV [112].

Another translocated deubiquitinase, RavD (Lpg0160) has been recently described [117]. This deubiquitinase differs from LotA and LotB in that it catalyzes the cleavage of linear polyubiquitin chains present on the LCV but not conventional branched isopeptide-linked polyubiquitin chains in mammalian cells [117]. In mammalian cells, the functional consequence of this deubiquitinase activity appears to be interference with the NF-κB signaling pathway during infection (Figure 3B) [117]. However, the natural amoebal hosts of *L. pneumophila* do not possess the NF-κB signaling pathway, suggesting the host target of RavD may still to be determined. Another study has implicated a role of RavD in diverting maturation of the LCV away from the endolysosomal pathway (Figure 3B) [118] through an unknown mechanism. It is possible RavD deubiquitinates proteins involved in evasion of the endolysosomal pathway to help establish the LCV in amoebae.

## 7. Conclusions

*L. pneumophila* has evolved within a diverse array of amoebal hosts to manipulate the eukaryotic ubiquitination machinery and does this by injecting effectors that mimic most known classes of ubiquitin ligases. Additionally, *L. pneumophila* has acquired novel ubiquitin ligases that mediate phosphoribosyl-ubiquitination of target proteins, independent of the canonical E1–E2 cascade, expanding the diversity of ubiquitin biology and shed new light on the evolution of ubiquitin-modifying enzymes. The discovery of novel ubiquitin ligases highlights the diverse array of *L. pneumophila* effectors that modulate the ubiquitin pathway, and furthermore, suggests potential amoebal origins through interkingdom gene transfer of novel ubiquitin-modifying enzymes. It is important to note that the target proteins and functional consequences of many of these effectors are currently unknown, and this may be due to most studies being confined to examining the role of these proteins in mammalian cells and not the natural amoebal hosts. This is not surprising since studying the cell biology of various unicellular eukaryotes lacks numerous biochemical and genetic tools available to study mammalian cells. However, *L. pneumophila* has evolved to survive and proliferate in a diverse range of amoebal species, and therefore it will be important to examine how these effectors have evolved to manipulate the ubiquitination pathway in the natural amoebae host. This will likely identify the true targets of these effectors, and also reveal novel ubiquitin biology in unicellular eukaryotes that can be applied to higher eukaryotes.

## Data Availability

All data presented in this review is freely available.

## References

[1] Vere G., Kealy R., Kessler B.M., Pinto-Fernandez A. (2020). Ubiquitomics: An Overview and Future. Biomolecules.

[2] Kliza K., Husnjak K. (2020). Resolving the Complexity of Ubiquitin Networks. Front. Mol. Biosci..

[3] Song L., Luo Z.-Q. (2019). Post-translational regulation of ubiquitin signaling. J. Cell Biol..

[4] Grice G.L., Nathan J.A. (2016). The recognition of ubiquitinated proteins by the proteasome. Cell. Mol. Life Sci..

[5] Kudriaeva A.A., Belogurov A.A. (2019). Proteasome: A Nanomachinery of Creative Destruction. Biochemistry.

[6] Schaefer A., Nethe M., Hordijk P.L. (2012). Ubiquitin links to cytoskeletal dynamics, cell adhesion and migration. Biochem. J..

[7] Aquila L., Atanassov B.S. (2020). Regulation of Histone Ubiquitination in Response to DNA Double Strand Breaks. Cells.

[8] Wang Y., Argiles-Castillo D., Kane E.I., Zhou A., Spratt D.E. (2020). HECT E3 ubiquitin ligases—emerging insights into their biological roles and disease relevance. J. Cell Sci..

[9] Leseigneur C., Lê-Bury P., Pizarro-Cerdá J., Dussurget O. (2020). Emerging Evasion Mechanisms of Macrophage Defenses by Pathogenic Bacteria. Front. Cell. Infect. Microbiol..

[10] Sachdeva K., Sundaramurthy V. (2020). The Interplay of Host Lysosomes and Intracellular Pathogens. Front. Cell. Infect. Microbiol..

[11] Kubelkova K., Macela A. (2019). Innate Immune Recognition: An Issue More Complex Than Expected. Front. Cell. Infect. Microbiol..

[12] Curto P., Riley S.P., Simões I., Martinez J.J. (2019). Macrophages Infected by a Pathogen and a Non-pathogen Spotted Fever Group Rickettsia Reveal Differential Reprogramming Signatures Early in Infection. Front. Cell. Infect. Microbiol..

[13] Faris R., Andersen S.E., McCullough A., Gourronc F., Klingelhutz A.J., Weber M.M. (2019). Chlamydia trachomatis Serovars Drive Differential Production of Proinflammatory Cytokines and Chemokines Depending on the Type of Cell Infected. Front. Cell. Infect. Microbiol..

[14] Thomas D.R., Newton P., Lau N., Newton H.J. (2020). Interfering with Autophagy: The Opposing Strategies Deployed by *Legionella pneumophila* and *Coxiella burnetii* Effector Proteins. Front. Cell. Infect. Microbiol..

[15] Snäkä T., Fasel N. (2020). Behind the Scenes: Nod-Like Receptor X1 Controls Inflammation and Metabolism. Front. Cell. Infect. Microbiol..

[16] Mambu J., Barilleau E., Fragnet-Trapp L., Le Vern Y., Olivier M., Sadrin G., Grépinet O., Taieb F., Velge P., Wiedemann A. (2020). Rck of *Salmonella typhimurium* Delays the Host Cell Cycle to Facilitate Bacterial Invasion. Front. Cell. Infect. Microbiol..

[17] Maurya R.K., Bharti S., Krishnan M.Y. (2019). Triacylglycerols: Fuelling the Hibernating *Mycobacterium tuberculosis*. Front. Cell. Infect. Microbiol..

[18] Del Portillo P., García-Morales L., Menéndez M.C., Anzola J.M., Rodríguez J.G., Helguera-Repetto A.C., Ares M.A., Prados-Rosales R., Gonzalez-Y-Merchand J.A., García M.J. (2019). Hypoxia Is Not a Main Stress When *Mycobacterium tuberculosis* Is in a Dormancy-Like Long-Chain Fatty Acid Environment. Front. Cell. Infect. Microbiol..

[19] Augenstreich J., Briken V. (2020). Host Cell Targets of Released Lipid and Secreted Protein Effectors of *Mycobacterium tuberculosis*. Front. Cell. Infect. Microbiol..

[20] Arora S.K., Naqvi N., Alam A., Ahmad J., Alsati B.S., Sheikh J.A., Kumar P., Mitra D.K., Rahman S.A., Hasnain S.E. (2020). *Mycobacterium smegmatis* Bacteria Expressing *Mycobacterium tuberculosis*-Specific Rv1954A Induce Macrophage Activation and Modulate the Immune Response. Front. Cell. Infect. Microbiol..

[21] Garg R., Borbora S.M., Bansia H., Rao S., Singh P., Verma R., Balaji K.N., Nagaraja V. (2020). *Mycobacterium tuberculosis* Calcium Pump CtpF Modulates the Autophagosome in an mTOR-Dependent Manner. Front. Cell. Infect. Microbiol..

[22] Çakır T., Panagiotou G., Uddin R., Durmuş S. (2020). Novel Approaches for Systems Biology of Metabolism-Oriented Pathogen-Human Interactions: A Mini-Review. Front. Cell. Infect. Microbiol..

[23] Price C.T.D., Al-Quadan T., Santic M., Rosenshine I., Abu Kwaik Y. (2011). Host Proteasomal Degradation Generates Amino Acids Essential for Intracellular Bacterial Growth. Science.

[24] Mohareer K., Medikonda J., Vadankula G.R., Banerjee S. (2020). Mycobacterial Control of Host Mitochondria: Bioenergetic and Metabolic Changes Shaping Cell Fate and Infection Outcome. Front. Cell. Infect. Microbiol..

[25] Chatterjee R., Chowdhury A.R., Mukherjee D., Chakravortty D. (2021). Lipid larceny: Channelizing host lipids for establishing successful pathogenesis by bacteria. Virulence.

[26] Kamanova J. (2020). Bordetella Type III Secretion Injectosome and Effector Proteins. Front. Cell. Infect. Microbiol..

[27] Wang X., Sun J., Wan L., Yang X., Lin H., Zhang Y., He X., Zhong H., Guan K., Min M. (2020). The Shigella Type III Secretion Effector IpaH4.5 Targets NLRP3 to Activate Inflammasome Signaling. Front. Cell. Infect. Microbiol..

[28] Gan J., Scott N.E., Newson J.P.M., Wibawa R.R., Lung T.W.F., Pollock G.L., Ng G.Z., Van Driel I., Pearson J.S., Hartland E.L. (2020). The *Salmonella* Effector SseK3 Targets Small Rab GTPases. Front. Cell. Infect. Microbiol..

[29] Feria J.M., Valvano M.A. (2020). An Overview of Anti-Eukaryotic T6SS Effectors. Front. Cell. Infect. Microbiol..

[30] Lou L., Zhang P., Piao R., Wang Y. (2019). *Salmonella* Pathogenicity Island 1 (SPI-1) and Its Complex Regulatory Network. Front. Cell. Infect. Microbiol..

[31] Green R.S., Izac J.R., Naimi W.A., O’Bier N., Breitschwerdt E.B., Marconi R.T., Carlyon J.A. (2020). *Ehrlichia chaffeensis* EplA Interaction with Host Cell Protein Disulfide Isomerase Promotes Infection. Front. Cell. Infect. Microbiol..

[32] Pan X., Luo J., Li S. (2020). Bacteria-Catalyzed Arginine Glycosylation in Pathogens and Host. Front. Cell. Infect. Microbiol..

[33] Herhaus L., Dikic I. (2018). Regulation of Salmonella-host cell interactions via the ubiquitin system. Int. J. Med Microbiol..

[34] Chai Q., Wang L., Liu C.H., Ge B. (2020). New insights into the evasion of host innate immunity by *Mycobacterium tuberculosis*. Cell. Mol. Immunol..

[35] Tanner K., Brzovic P., Rohde J.R. (2014). The bacterial pathogen-ubiquitin interface: Lessons learned from Shigella. Cell. Microbiol..

[36] Qiu J., Luo Z.-Q. (2017). *Legionella* and *Coxiella* effectors: Strength in diversity and activity. Nat. Rev. Genet..

[37] Huang B., Ojogun N., Ragland S.A., Carlyon J.A. (2012). Monoubiquitinated proteins decorate the Anaplasma phagocytophilum-occupied vacuolar membrane. FEMS Immunol. Med. Microbiol..

[38] Zhou Y., Zhu Y. (2015). Diversity of bacterial manipulation of the host ubiquitin pathways. Cell. Microbiol..

[39] Hayward R.J., Marsh J.W., Humphrys M.S., Huston W.M., Myers G.S.A. (2019). Early Transcriptional Landscapes of *Chlamydia trachomatis*-Infected Epithelial Cells at Single Cell Resolution. Front. Cell. Infect. Microbiol..

[40] Kunz T.C., Götz R., Sauer M., Rudel T. (2019). Detection of Chlamydia Developmental Forms and Secreted Effectors by Expansion Microscopy. Front. Cell. Infect. Microbiol..

[41] Bartra S.S., Lorica C., Qian L., Gong X., Bahnan W., Barreras H.B., Hernandez R., Li Z., Plano G.V., Schesser K. (2019). Chromosomally-Encoded *Yersinia pestis* Type III Secretion Effector Proteins Promote Infection in Cells and in Mice. Front. Cell. Infect. Microbiol..

[42] Kitao T., Nagai H., Kubori T. (2020). Divergence of *Legionella* Effectors Reversing Conventional and Unconventional Ubiquitination. Front. Cell. Infect. Microbiol..

[43] Best A., Abu Kwaik Y. (2018). Evolution of the Arsenal of *Legionella pneumophila* Effectors to Modulate Protist Hosts. mBio.

[44] Gomez-Valero L., Buchrieser C. (2019). Intracellular parasitism, the driving force of evolution of *Legionella pneumophila* and the genus *Legionella*. Genes Immun..

[45] Mondino S., Schmidt S., Buchrieser C. (2020). Molecular Mimicry: A Paradigm of Host-Microbe Coevolution Illustrated by *Legionella*. mBio.

[46] Li P., Vassiliadis D., Ong S.Y., Bennett-Wood V., Sugimoto C., Yamagishi J., Hartland E.L., Pasricha S. (2020). *Legionella pneumophila* Infection Rewires the *Acanthamoeba castellanii* Transcriptome, Highlighting a Class of Sirtuin Genes. Front. Cell. Infect. Microbiol..

[47] Fields B.S. (1996). The molecular ecology of legionellae. Trends Microbiol..

[48] Harb O.S., Gao L.-Y., Abu Kwaik Y. (2000). From protozoa to mammalian cells: A new paradigm in the life cycle of intracellular bacterial pathogens. Minireview. Environ. Microbiol..

[49] Molmeret M., Horn M., Wagner M., Santic M., Abu Kwaik Y. (2005). Amoebae as Training Grounds for Intracellular Bacterial Pathogens. Appl. Environ. Microbiol..

[50] Haenssler E., Ramabhadran V., Murphy C.S., Heidtman M.I., Isberg R.R. (2015). Endoplasmic Reticulum Tubule Protein Reticulon 4 Associates with the *Legionella pneumophila* Vacuole and with Translocated Substrate Ceg9. Infect. Immun..

[51] Isberg R.R., O’Connor T.J., Heidtman M. (2008). The *Legionella pneumophila* replication vacuole: Making a cosy niche inside host cells. Nat. Rev. Genet..

[52] Kagan J.C., Roy C.R. (2002). *Legionella* phagosomes intercept vesicular traffic from endoplasmic reticulum exit sites. Nat. Cell Biol..

[53] Younes S., Al-Sulaiti A., Nasser E.A.A., Najjar H., Kamareddine L. (2020). Drosophila as a Model Organism in Host–Pathogen Interaction Studies. Front. Cell. Infect. Microbiol..

[54] Horwitz M.A. (1984). Phagocytosis of the legionnaires’ disease bacterium (*legionella pneumophila*) occurs by a novel mechanism: Engulfment within a Pseudopod coil. Cell.

[55] Burstein D., Amaro F., Zusman T., Lifshitz Z., Cohen O., Gilbert J.A., Pupko T., Shuman H.A., Segal G. (2016). Genomic analysis of 38 *Legionella* species identifies large and diverse effector repertoires. Nat. Genet..

[56] Zhu W., Banga S., Tan Y., Zheng C., Stephenson R., Gately J., Luo Z.-Q. (2011). Comprehensive Identification of Protein Substrates of the Dot/Icm Type IV Transporter of Legionella pneumophila. PLoS ONE.

[57] Price C.T., Al-Khodor S., Al-Quadan T., Santic M., Habyarimana F., Kalia A., Abu Kwaik Y. (2009). Molecular Mimicry by an F-Box Effector of *Legionella pneumophila* Hijacks a Conserved Polyubiquitination Machinery within Macrophages and Protozoa. PLOS Pathog..

[58] Horwitz M.A. (1983). The Legionnaires’ disease bacterium (*Legionella pneumophila*) inhibits phagosome-lysosome fusion in human monocytes. J. Exp. Med..

[59] Horwitz M.A. (1983). Formation of a novel phagosome by the Legionnaires’ disease bacterium (*Legionella pneumophila*) in human monocytes. J. Exp. Med..

[60] Horwitz M.A., Silverstein S.C. (1980). Legionnaires’ Disease Bacterium (*Legionella pneumophila*) Multiplies Intracellularly in Human Monocytes. J. Clin. Investig..

[61] Al-Khodor S., Price C.T., Habyarimana F., Kalia A., Abu Kwaik Y. (2008). A Dot/Icm-translocated ankyrin protein of *Legionella pneumophila* is required for intracellular proliferation within human macrophages and protozoa. Mol. Microbiol..

[62] Ensminger A.W., Isberg R.R. (2010). E3 Ubiquitin Ligase Activity and Targeting of BAT3 by Multiple *Legionella pneumophila* Translocated Substrates. Infect. Immun..

[63] Nguyen K.M., Busino L. (2020). The Biology of F-box Proteins: The SCF Family of E3 Ubiquitin Ligases. Adv. Exp. Med. Biol..

[64] Tekcham D.S., Chen D., Liu Y., Ling T., Zhang Y., Chen H., Wang W., Otkur W., Qi H., Xia T. (2020). F-box proteins and cancer: An update from functional and regulatory mechanism to therapeutic clinical prospects. Theranostics.

[65] Price C.T.D., Abu Kwaik Y. (2010). Exploitation of Host Polyubiquitination Machinery through Molecular Mimicry by Eukaryotic-Like Bacterial F-Box Effectors. Front. Microbiol..

[66] Lomma M., Dervins-Ravault D., Rolando M., Nora T., Newton H.J., Sansom F.M., Sahr T., Gomez-Valero L., Jules M., Hartland E.L. (2010). The *Legionella pneumophila* F-box protein Lpp2082 (AnkB) modulates ubiquitination of the host protein parvin B and promotes intracellular replication. Cell. Microbiol..

[67] Price C.T., Al-Quadan T., Santic M., Jones S.C., Abu Kwaik Y. (2010). Exploitation of conserved eukaryotic host cell farnesylation machinery by an F-box effector of *Legionella pneumophila*. J. Exp. Med..

[68] Wong K., Perpich J.D., Kozlov G., Cygler M., Abu Kwaik Y., Gehring K. (2017). Structural Mimicry by a Bacterial F Box Effector Hijacks the Host Ubiquitin-Proteasome System. Structure.

[69] Price C.T.D., Richards A.M., Von Dwingelo J.E., Samara H.A., Abu Kwaik Y. (2013). Amoeba host-*Legionella* synchronization of amino acid auxotrophy and its role in bacterial adaptation and pathogenic evolution. Environ. Microbiol..

[70] Jiang H., Zhang X., Chen X., Aramsangtienchai P., Tong Z., Lin H. (2018). Protein Lipidation: Occurrence, Mechanisms, Biological Functions, and Enabling Technologies. Chem. Rev..

[71] Perpich J.D., Kalia A., Price C.T.D., Jones S.C., Wong K., Gehring K., Abu Kwaik Y. (2017). Divergent evolution of Di-lysine ER retention vs. farnesylation motif-mediated anchoring of the AnkB virulence effector to the *Legionella*-containing vacuolar membrane. Sci. Rep..

[72] Price C.T.D., Merchant M., Jones S., Best A., Von Dwingelo J., Lawrenz M.B., Alam N., Schueler-Furman O., Abu Kwaik Y. (2017). Host FIH-Mediated Asparaginyl Hydroxylation of Translocated *Legionella pneumophila* Effectors. Front. Cell. Infect. Microbiol..

[73] Strowitzki M.J., Cummins E.P., Taylor C.T. (2019). Protein Hydroxylation by Hypoxia-Inducible Factor (HIF) Hydroxylases: Unique or Ubiquitous?. Cells.

[74] Bruckert W.M., Abu Kwaik Y. (2015). Lysine11-Linked Polyubiquitination of the AnkB F-Box Effector of *Legionella pneumophila*. Infect. Immun..

[75] Rothmeier E., Pfaffinger G., Hoffmann C., Harrison C.F., Grabmayr H., Repnik U., Hannemann M., Wölke S., Bausch A., Griffiths G. (2013). Activation of Ran GTPase by a *Legionella* Effector Promotes Microtubule Polymerization, Pathogen Vacuole Motility and Infection. PLOS Pathog..

[76] Lee J.-G., Ye Y. (2013). Bag6/Bat3/Scythe: A novel chaperone activity with diverse regulatory functions in protein biogenesis and degradation. BioEssays.

[77] Ivanov S.S., Charron G., Hang H.C., Roy C.R. (2010). Lipidation by the Host Prenyltransferase Machinery Facilitates Membrane Localization of *Legionella pneumophila* Effector Proteins. J. Biol. Chem..

[78] Kubori T., Hyakutake A., Nagai H. (2008). *Legionella* translocates an E3 ubiquitin ligase that has multiple U-boxes with distinct functions. Mol. Microbiol..

[79] Kubori T., Shinzawa N., Kanuka H., Nagai H. (2010). *Legionella* Metaeffector Exploits Host Proteasome to Temporally Regulate Cognate Effector. PLOS Pathog..

[80] Lin Y.-H., Lucas M., Evans T.R., Abascal-Palacios G., Doms A.G., Beauchene N.A., Rojas A.L., Hierro A., Machner M.P. (2018). RavN is a member of a previously unrecognized group of *Legionella pneumophila* E3 ubiquitin ligases. PLOS Pathog..

[81] Lin Y.-H., Doms A.G., Cheng E., Kim B., Evans T.R., Machner M.P. (2015). Host Cell-catalyzed S-Palmitoylation Mediates Golgi Targeting of the *Legionella* Ubiquitin Ligase GobX. J. Biol. Chem..

[82] Hatakeyama S., Nakayama K.-I. (2003). U-box proteins as a new family of ubiquitin ligases. Biochem. Biophys. Res. Commun..

[83] Quaile A.T., Urbanus M.L., Stogios P.J., Nocek B., Skarina T., Ensminger A.W., Savchenko A. (2015). Molecular Characterization of LubX: Functional Divergence of the U-Box Fold by *Legionella pneumophila*. Structure.

[84] Urbanus M.L., Quaile A.T., Stogios P.J., Morar M., Rao C., Di Leo R., Evdokimova E., Lam M., Oatway C., Cuff M.E. (2016). Diverse mechanisms of metaeffector activity in an intracellular bacterial pathogen, *Legionella pneumophila*. Mol. Syst. Biol..

[85] Ragaz C., Pietsch H., Urwyler S., Tiaden A., Weber S.S., Hilbi H. (2008). The *Legionella pneumophila* phosphatidylinositol-4 phosphate-binding type IV substrate SidC recruits endoplasmic reticulum vesicles to a replication-permissive vacuole. Cell. Microbiol..

[86] Brombacher E., Urwyler S., Ragaz C., Weber S.S., Kami K., Overduin M., Hilbi H. (2009). Rab1 Guanine Nucleotide Exchange Factor SidM Is a Major Phosphatidylinositol 4-Phosphate-binding Effector Protein of *Legionella pneumophila*. J. Biol. Chem..

[87] Horenkamp F.A., Mukherjee S., Alix E., Schauder C.M., Hubber A.M., Roy C.R., Reinisch K.M. (2014). *Legionella pneumophila* Subversion of Host Vesicular Transport by SidC Effector Proteins. Traffic.

[88] Dolinsky S., Haneburger I., Cichy A., Hannemann M., Itzen A., Hilbi H. (2014). The *Legionella* longbeachae Icm/Dot Substrate SidC Selectively Binds Phosphatidylinositol 4-Phosphate with Nanomolar Affinity and Promotes Pathogen Vacuole-Endoplasmic Reticulum Interactions. Infect. Immun..

[89] Luo X., Wasilko D.J., Liu Y., Sun J., Wu X., Luo Z.-Q., Mao Y. (2015). Structure of the *Legionella* Virulence Factor, SidC Reveals a Unique PI(4)P-Specific Binding Domain Essential for Its Targeting to the Bacterial Phagosome. PLOS Pathog..

[90] Hsu F., Luo X., Qiu J., Teng Y.-B., Jin J., Smolka M.B., Luo Z.-Q., Mao Y. (2014). The *Legionella* effector SidC defines a unique family of ubiquitin ligases important for bacterial phagosomal remodeling. Proc. Natl. Acad. Sci. USA.

[91] Wasilko D.J., Huang Q., Mao Y. (2018). Insights into the ubiquitin transfer cascade catalyzed by the *Legionella* effector SidC. eLife.

[92] Jeng E.E., Bhadkamkar V., Ibe N.U., Gause H., Jiang L., Chan J., Jian R., Jimenez-Morales D., Stevenson E., Krogan N.J. (2019). Systematic Identification of Host Cell Regulators of *Legionella pneumophila* Pathogenesis Using a Genome-wide CRISPR Screen. Cell Host Microbe.

[93] Qiu J., Sheedlo M.J., Yu K., Tan Y., Nakayasu E.S., Das C., Liu X., Luo Z.Q. (2016). Ubiquitination independent of E1 and E2 enzymes by bacterial effectors. Nature.

[94] Bhogaraju S., Kalayil S., Liu Y., Bonn F., Colby T., Matic I., Dikic I. (2016). Phosphoribosylation of Ubiquitin Promotes Serine Ubiquitination and Impairs Conventional Ubiquitination. Cell.

[95] Wang Y., Shi M., Feng H., Zhu Y., Liu S., Gao A., Gao P. (2018). Structural Insights into Non-canonical Ubiquitination Catalyzed by SidE. Cell.

[96] Dong Y., Mu Y., Xie Y., Zhang Y., Han Y., Zhou Y., Wang W., Liu Z., Wu M., Wang H. (2018). Structural basis of ubiquitin modification by the *Legionella* effector SdeA. Nature.

[97] Akturk A., Wasilko D.J., Wu X., Liu Y., Zhang Y., Qiu J., Luo Z.-Q., Reiter K.H., Brzovic P.S., Klevit R.E. (2018). Mechanism of phosphoribosyl-ubiquitination mediated by a single *Legionella* effector. Nature.

[98] Kalayil S., Bhogaraju S., Bonn F., Shin D., Liu Y., Gan N., Basquin J., Grumati P., Luo Z.-Q., Dikic I. (2018). Insights into catalysis and function of phosphoribosyl-linked serine ubiquitination. Nature.

[99] Kim L., Kwon D.H., Kim B.H., Kim J., Park M.R., Park Z.-Y., Song H.K. (2018). Structural and Biochemical Study of the Mono-ADP-Ribosyltransferase Domain of SdeA, a Ubiquitylating/Deubiquitylating Enzyme from *Legionella pneumophila*. J. Mol. Biol..

[100] Kotewicz K.M., Ramabhadran V., Sjoblom N., Vogel J.P., Haenssler E., Zhang M., Behringer J., Scheck R.A., Isberg R.R. (2017). A Single *Legionella* Effector Catalyzes a Multistep Ubiquitination Pathway to Rearrange Tubular Endoplasmic Reticulum for Replication. Cell Host Microbe.

[101] Havey J.C., Roy C.R. (2015). Toxicity and SidJ-Mediated Suppression of Toxicity Require Distinct Regions in the SidE Family of *Legionella pneumophila* Effectors. Infect. Immun..

[102] Jeong K.C., Sexton J.A., Vogel J.P. (2015). Spatiotemporal Regulation of a *Legionella pneumophila* T4SS Substrate by the Metaeffector SidJ. PLoS Pathog..

[103] Qiu J., Yu K., Fei X., Liu Y., Nakayasu E.S., Piehowski P.D., Shaw J.B., Puvar K., Das C., Liu X. (2017). A unique deubiquitinase that deconjugates phosphoribosyl-linked protein ubiquitination. Cell Res..

[104] Black M.H., Osinski A., Gradowski M., Servage K., Pawłowski K., Tomchick D.R., Tagliabracci V. (2019). Bacterial pseudokinase catalyzes protein polyglutamylation to inhibit the SidE-family ubiquitin ligases. Science.

[105] Gan N., Zhen X., Liu Y., Xu X., He C., Qiu J., Liu Y., Fujimoto G.M., Nakayasu E.S., Zhou B. (2019). Regulation of phosphoribosyl ubiquitination by a calmodulin-dependent glutamylase. Nature.

[106] Bhogaraju S., Bonn F., Mukherjee R., Adams M., Pfleiderer M.M., Galej W.P., Matkovic V., Lopez-Mosqueda J., Kalayil S., Shin D. (2019). Inhibition of bacterial ubiquitin ligases by SidJ–calmodulin catalysed glutamylation. Nature.

[107] Sulpizio A., Minelli M.E., Wan M., Burrowes P.D., Wu X., Sanford E.J., Shin J.-H., Williams B.C., Goldberg M.L., Smolka M.B. (2019). Protein polyglutamylation catalyzed by the bacterial calmodulin-dependent pseudokinase SidJ. eLife.

[108] Wan M., Sulpizio A.G., Akturk A., Beck W.H.J., Lanz M., Faça V.M., Smolka M.B., Vogel J.P., Mao Y. (2019). Deubiquitination of phosphoribosyl-ubiquitin conjugates by phosphodiesterase-domain–containing *Legionella* effectors. Proc. Natl. Acad. Sci. USA.

[109] Shin D., Mukherjee R., Liu Y., Gonzalez A., Bonn F., Liu Y., Rogov V.V., Heinz M., Stolz A., Hummer G. (2020). Regulation of Phosphoribosyl-Linked Serine Ubiquitination by Deubiquitinases DupA and DupB. Mol. Cell.

[110] Kubori T., Kitao T., Ando H., Nagai H. (2018). LotA, a *Legionella* deubiquitinase, has dual catalytic activity and contributes to intracellular growth. Cell. Microbiol..

[111] Ma K., Zhen X., Zhou B., Gan N., Cao Y., Fan C., Ouyang S., Luo Z.-Q., Qiu J. (2020). The bacterial deubiquitinase Ceg23 regulates the association of Lys-63–linked polyubiquitin molecules on the *Legionella* phagosome. J. Biol. Chem..

[112] Kitao T., Taguchi K., Seto S., Arasaki K., Ando H., Nagai H., Kubori T. (2020). *Legionella* Manipulates Non-canonical SNARE Pairing Using a Bacterial Deubiquitinase. Cell Rep..

[113] Risselada H., Mayer A. (2020). SNAREs, tethers and SM proteins: How to overcome the final barriers to membrane fusion?. Biochem. J..

[114] Derré I., Isberg R.R. (2004). *Legionella pneumophila* Replication Vacuole Formation Involves Rapid Recruitment of Proteins of the Early Secretory System. Infect. Immun..

[115] Kagan J.C., Stein M.-P., Pypaert M., Roy C.R. (2004). *Legionella* Subvert the Functions of Rab1 and Sec22b to Create a Replicative Organelle. J. Exp. Med..

[116] Arasaki K., Roy C.R. (2010). *Legionella pneumophila* Promotes Functional Interactions between Plasma Membrane Syntaxins and Sec22b. Traffic.

[117] Wan M., Wang X., Huang C., Xu D., Wang Z., Zhou Y., Zhu Y. (2019). A bacterial effector deubiquitinase specifically hydrolyses linear ubiquitin chains to inhibit host inflammatory signalling. Nat. Microbiol..

[118] Pike C.M., Boyer-Andersen R., Kinch L.N., Caplan J.L., Neunuebel M.R. (2019). The *Legionella* effector RavD binds phosphatidylinositol-3-phosphate and helps suppress endolysosomal maturation of the *Legionella*-containing vacuole. J. Biol. Chem..

